# Good versus poor prescribers: the comparison of prescribing competencies in primary care

**DOI:** 10.1017/S1463423622000111

**Published:** 2022-03-28

**Authors:** Omer Atac, Volkan Aydin, Selma Karabey, Osman Hayran, Ahmet Akici

**Affiliations:** 1 Department of Public Health, School of Medicine, Istanbul Medipol University, Istanbul, Turkey; 2 Department of Medical Pharmacology, International School of Medicine, Istanbul Medipol University, Istanbul, Turkey; 3 Department of Public Health, Istanbul Faculty of Medicine, Istanbul University, Istanbul, Turkey; 4 Department of Medical Pharmacology, School of Medicine, Marmara University, Istanbul, Turkey

**Keywords:** diagnosis, pharmacotherapy, physicians, prescribing performance, primary care, rational use of medicine

## Abstract

**Aim:**

To compare the competencies of primary care physicians (PCPs) with poor and good prescribing performance in frequently encountered indications.

**Background:**

Primary care centers are one of the mostly visited health facilities by the population for different health issues.

**Methods:**

In this cross-sectional study, we analyzed 6 125 487 prescriptions generated by 1431 PCPs which were selected by systematic sampling in 2016 in Istanbul. We defined PCPs as poor prescriber (n = 227) or good prescriber (n = 210) in terms of their prescribing performance per WHO/INRUD criteria. We compared solo diagnosis prescriptions of these two groups in ‘percentage of prescriptions in compliance with clinical guidelines’ and also rational prescribing indicators.

**Findings:**

Poor prescribers and good prescribers significantly differed in each of the prescribing indicators for their all solo diagnosis prescriptions. Hypertension had the highest difference of the average cost per encounter (Δ = 284.2%) between poor prescribers (US$43.99 ± 63.05) and good prescribers (US$11.45 ± 45.0), whereas headache had the highest difference between the groups in the percentage encounters with an antibiotic (14.9% vs. 1.5%). Detailed analysis of the prescribing performances showed significantly higher values of each WHO/INRUD indicators for all examined diagnoses. We found significantly higher percentages of guideline-compliant drugs in good prescribers compared to that in poor prescribers in hypertension (40.8% vs 34.8%), tonsillopharyngitis (57.9% vs 50.7%), and acute sinusitis (46.4% vs 43.6%).

**Conclusion:**

This study shows that the prescribing performances of PCPs are not rational enough in terms of drug selection and prescription content. Furthermore, even the physicians who have good prescribing practice appear as not satisfactorily rational in compliance with current pharmacotherapy competencies.

## Introduction

Primary care centers are the mostly consulted health facilities by the community for seeking medical advice, getting a diagnosis, or initiating or maintaining treatment. Although the organization and scope of these services vary among countries, it is expected to be practiced as rational as possible for the sustainability of the health system. Rational use of medicine (RUM) is defined as “patients receive medications that are appropriate to their clinical needs, in doses that meet their own individual requirements, for an adequate period of time, and at the lowest cost to them and their community” (World Health Organization, 2002). Physicians are at the core of the efforts that help to ensure RUM practice because of their central role in establishing the diagnosis, planning the treatment, and prescribing. The failure of physicians to follow appropriate steps especially for treatment may cause serious problems of irrational use of medicine: use of drugs in inappropriate indications or discordant to treatment guidelines, tendency to polypharmacy, unnecessary/excessive use of antibiotics and injectable preparations, and inattention for costly medications (World Health Organization, 2002; 2004). Physicians’ daily workloads such as direct patient contacts, follow-ups, counseling, consultations, and home visits may also affect their prescribing performance (Granja *et al.*, 2014).

Despite its practical difficulties, an evidence-based approach is expected to be applied with updated medical information during prescribing. Guidelines prepared and updated by reliable authorities could be helpful in this context. Primary care physicians (PCPs) are expected to benefit from these guidelines to ease their practice since they deal with a large number of indications and drugs. On the other hand, prescriptions not complying with guidelines and algorithms can lead to patient harm, therapeutic failure, drug resistance, and waste of resources (World Health Organization, 2002). Apart from guideline adherence, other internationally accepted criteria are also used for evaluating RUM performances of physicians. The World Health Organization/International Network for the Rational Use of Drugs (WHO/INRUD) drug use indicators constitute a guidance for the assessment of physicians’ prescriptions in terms of RUM (World Health Organization, 1993).

In Turkey, PCPs received one-third of medical visits in the last five years (The Ministry of Health of Turkey, 2018). Prescriptions are generated electronically and can be monitored by the national health authority via the Prescription Information System (PIS) (Koyuncuoglu *et al.*, 2017). PCPs carry out different types of healthcare services including treatment of chronic diseases, e.g. hypertension, and acute conditions, e.g. infectious diseases (Chow *et al.*, 2012; Mancia *et al.*, 2013). Disclosing the performance of PCPs in prevalent conditions can accelerate the dissemination of RUM. In this study, we aimed to compare the competencies of PCPs with poor and good prescribing performance in frequently encountered indications.

## Methods

### Study type and sample size

In this cross-sectional study, we analyzed prescriptions generated by PCPs in 2016 in Istanbul where a total of 4293 primary care units were present. Istanbul is a metropolitan city with near 15 million inhabitants (18.5% of the country), who constitute 17.4% of all PCPs in the country (The Ministry of Health of Turkey, 2018; Turkish Statistical Institute, 2016). We calculated the minimum sample size of the study as at least 353 PCPs with 95% confidence level, 5% margin of error, and 50% response distribution (Raosoft, 2017). By systematic sampling, PCPs were ascendingly listed in terms of their anonymous ID. Then, we selected 1431 PCPs as one out of every three physicians and included in this study with their 6 125 487 prescriptions registered to PIS between 1January 2016 and 31 December 2016.

### Identification of diagnoses

As the prescriptions with multiple diagnoses are not eligible for assessing the appropriateness of a drug for a particular indication, we examined compliance to treatment guidelines in 2 947 112 (49.6%) prescriptions with solo diagnosis. We determined the diagnoses by considering their relative rank of frequency as solo diagnosis and whether it had a specific treatment guideline. Among the indications that are included in the top 10 frequent diagnoses, we identified ‘I10-essential hypertension’ (9.5%), ‘J02_J03-tonsillopharyngitis’ (6.6%), ‘J01-acute sinusitis’ (2.8%), and ‘Z00-general examination (1.7%)’, and non-primary care dental practice, ‘K02-dental caries’ (0.6%) as disease-based indications. We also identified substantial number of prescriptions that only contained a particular symptom in the diagnosis section. The five most common symptom-based diagnoses included ‘M54-dorsalgia’ (1.6%), ‘K30-functional dyspepsia’ (0.7%), ‘R05-cough’ (0.7%), ‘R11-nausea and vomiting’ (0.5%), and ‘R51-headache’ (0.4%).

### Identification of the PCP groups

**Figure 1.**
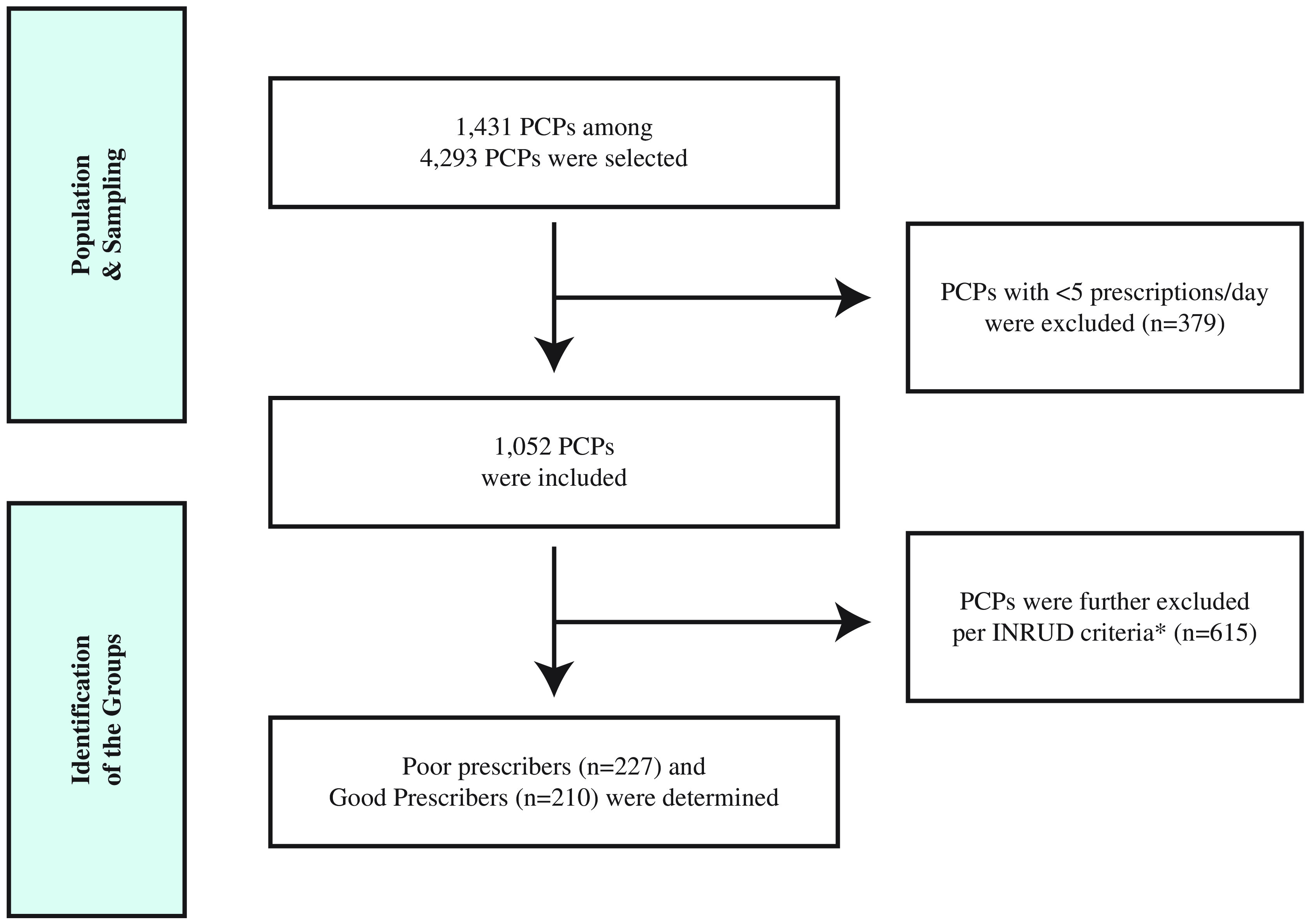
Selection cascade of the prescriber groups. *Poor and good prescribers represented at the highest and lowest quartile of primary care physicians in at least two of three selected WHO/INRUD criteria, respectively.

To determine the PCP groups whose prescribing performances would be compared, we first ranked all physicians in a descending manner based on the total number of prescriptions. Among these eligible 1052 PCPs, we further ranked these per each of the ‘average number of medicines per encounter’, ‘percentage of encounters with an antibiotic’, and ‘average medicine cost per encounter’ in descending order for all prescriptions (regardless of solo/multiple diagnosis). We defined a PCP as a poor prescriber if s/he was qualified to the highest quartile in at least two of these three WHO/INRUD criteria, or as a good prescriber if qualified to the lowest quartile in at least two of these criteria. These allowed us to assign 227 poor prescribers and 210 good prescribers (Figure 1). The distribution of the drug utilization criteria by the PCP quartiles was summarized in Table S1 for all prescriptions and Table S2 for solo diagnosis prescriptions in Supplementary Material.

### Study variables

We compared specific study-defined solo diagnosis prescriptions of these two PCP groups in terms of ‘average number of medicines per encounter’, ‘percentage of encounters with antibiotic’, ‘average medicine cost per encounter’, and ‘percentage of prescriptions in compliance with clinical guidelines’.

We determined the criteria for the assessment of compliance to guidelines by considering the most current versions regarding the selected diagnoses before the year 2016. We listed appropriate and contraindicated treatment recommendations for each selected diagnosis (Chow *et al.*, 2012; Mancia *et al.*, 2013; NICE, 2016; Rosenfeld *et al.*, 2015; SDCEP, 2011; Shulman *et al.*, 2012). The adoptability and appropriateness of these recommendations for primary care were supported by additional expert opinions through a standardized ‘expert opinion form’, assessed separately by a clinical pharmacologist, a family physician, and five other specialists relevant to the study-defined indications: an infectious diseases specialist, an otorhinolaryngologist, a cardiologist, a urologist, and two dentists. The forms were delivered face-to-face and/or via e-mail to academic specialists who were not included among the study group. After giving a certain period of time for their assessment, the signed forms were collected. The diagnoses selected for the assessment of adherence to guidelines were presented in each form regarding the specialties of the physicians. All diagnoses were included in the forms of the family physician and clinical pharmacologist. The forms contained the drug/drug groups are recommended as the first choice and contraindicated according to the guidelines as closed-end questions. In addition to this, an open-ended section was provided where specialists could write the additional recommendations in the forms. All forms were examined and finalized by the study investigators to verify and ensure that the expert opinions were consistent with the guidelines. In compliance with these expert opinions and treatment guidelines, we used the “lists for compliance to treatment guidelines” to assess the prescribing performance of the physicians.

As a critical WHO/INRUD indicator, use of antibiotic subgroups was further described in PCP prescriptions. In addition, some other drug groups commonly used in primary care were evaluated, including ‘non-steroidal anti-inflammatory drugs’ (NSAIDs; M01A), ‘cough suppressants (R05DB)’, and ‘proton-pump inhibitors’ (PPI; A02BC).

### Statistical analyses

We used Microsoft Office Excel 2016 and SPSS 24.0 for data analysis. Descriptive data were presented as the number, percentage, mean, and standard deviation, wherever appropriate. We compare the study groups via student t-test and Mann-Whitney U test for normally and non-normally distributed continuous variables, respectively; and chi-square test for categorical variables. An overall 5% of Type-I error level was used to infer statistical significance.

### Official approvals

Required official permissions were received to use the PIS data. The study was conducted after a protocol was signed between The Ministry of Health of Turkey and Istanbul Medipol University for a limited access to prescription data within the General Directorate of Health Information Systems. This study was approved by the Ethics Committee of Non-interventional Clinical Trials of Istanbul Medipol University.

## Results

Poor prescribers and good prescribers were found to generate 18.7% and %22.3 of solo diagnosis prescriptions with the average number of medicines as 2.38 ± 1.20 vs. 1.92 ± 0.24, respectively (Supplementary Table S2). The five disease-based and five symptom-based diagnoses constituted 26.9% and 23.3% of all solo diagnosis prescriptions of poor and good prescribers, respectively. All of the 10 diagnoses were among the top 50 indications written by physicians. Specifically, hypertension ranked first in poor prescribers and second in good prescribers, followed by tonsillopharyngitis in both groups (Supplementary Table S3).

Among the most frequently written 100 diagnoses for the study population, disease-based diagnoses constituted 94.7% in poor prescribers and 93.4% in good prescribers (*P* < 0.001). In these indications, poor prescribers were more likely to prescribe for communicable diseases (60.3%) than were good prescribers (49.6%, *P* < 0.001).

Prescribing performances of PCPs showed significantly higher values of each of WHO/INRUD indicators in poor prescribers vs. good prescribers for all disease-based and symptom-based diagnoses in the study (*P* < 0.001 for all pairwise comparisons). The average number of medicines per encounter ranged from 2.02 ± 1.00 (dental caries) to 3.17 ± 0.99 (dyspepsia) in poor prescribers, whereas this varied between 1.88 ± 0.90 (dental caries) and 2.52 ± 0.95 (acute sinusitis) in good prescribers. In this comparison, we detected the highest differences between the two PCP groups for headache (Δ = 88.8%), cough (Δ = 67.1%), and dyspepsia (Δ = 61.7%), (Table 1).

**Table 1.** The comparison of the performances of the study groups for selected WHO/INRUD drug-use indicators

		Poor prescribers	Good prescribers	**Δ** * **(%)**
**Disease-based diagnoses**				
**Hypertension**	Average number of medicines per encounter, n (±SD)	2.35 ± 1.87	1.96 ± 1.45	**19.9**
	Average medicine cost per encounter, US$ (±SD)	43.99 ± 63.05	11.45 ± 45.00	**284.2**
	Encounters with an antibiotic prescribed, %	3.2	1.1	**190.9**
**Tonsillopharyngitis**	Average number of medicines per encounter, n (±SD)	2.94 ± 1.49	2.44 ± 0.90	**20.5**
	Average medicine cost per encounter, US$ (±SD)	11.47 ± 26.05	7.28 ± 6.36	**57.6**
	Encounters with an antibiotic prescribed, %	75.3	73.9	**1.9**
**Acute sinusitis**	Average number of medicines per encounter, n (±SD)	3.06 ± 1.31	2.52 ± 0.95	**21.4**
	Average medicine cost per encounter, US$ (±SD)	13.11 ± 20.69	8.40 ± 6.94	**56.1**
	Encounters with an antibiotic prescribed, %	86.8	79.9	**8.6**
**Dental caries**	Average number of medicines per encounter, n (±SD)	2.02 ± 1.00	1.88 ± 0.90	**7.5**
	Average medicine cost per encounter, US$ (±SD)	8.91 ± 16.68	6.85 ± 16.59	**30.1**
	Encounters with an antibiotic prescribed, %	91.8	86.0	**6.7**
**General examination**	Average number of medicines per encounter, n (±SD)	2.76 ± 2.02	2.17 ± 1.59	**27.2**
	Average medicine cost per encounter, US$ (±SD)	47.13 ± 158.47	20.95 ± 5.15	**125.0**
	Encounters with an antibiotic prescribed, %	23.1	9.0	**156.7**
**Symptom-based diagnoses**				
**Dyspepsia**	Average number of medicines per encounter, n (±SD)	3.17 ± 0.99	1.96 ± 0.64	**61.7**
	Average medicine cost per encounter, US$ (±SD)	40.39 ± 26.91	16.80 ± 12.44	**140.4**
	Encounters with an antibiotic prescribed, %	6.1	2.8	**117.9**
**Dorsalgia**	Average number of medicines per encounter, n (±SD)	2.48 ± 0.50	2.07 ± 0.32	**19.8**
	Average medicine cost per encounter, US$ (±SD)	14.21 ± 7.09	9.51 ± 6.14	**49.4**
	Encounters with an antibiotic prescribed, %	1.1	0.5	**120.0**
**Cough**	Average number of medicines per encounter, n (±SD)	2.94 ± 1.15	1.76 ± 0.53	**67.1**
	Average medicine cost per encounter, US$ (±SD)	19.94 ± 11.16	9.13 ± 6.48	**118.4**
	Encounters with an antibiotic prescribed, %	25.3	7.9	**220.3**
**Nausea and vomitting**	Average number of medicines per encounter, n (±SD)	2.60 ± 1.17	1.64 ± 0.49	**58.5**
	Average medicine cost per encounter, US$ (±SD)	16.41 ± 19.85	5.25 ± 4.87	**212.6**
	Encounters with an antibiotic prescribed, %	18.7	5.0	**274.0**
**Headache**	Average number of medicines per encounter, n (±SD)	3.02 ± 0.95	1.60 ± 0.55	**88.8**
	Average medicine cost per encounter, US$ (±SD)	31.50 ± 18.10	11.83 ± 11.57	**166.3**
	Encounters with an antibiotic prescribed, %	14.9	1.5	**899.3**

* Delta calculation was made by considering good prescribers’ data compared to poor prescribers’. SD, standard deviation.

The average medicine cost per encounter was the highest in ‘general examination’ prescriptions for both poor prescribers (US $47.13 ± 158.47) and good prescribers (US $20.95 ± 5.15). Hypertension was the diagnosis with the highest difference of the average cost per encounter (Δ = 284.2%) between poor prescribers (US $43.99 ± 63.05) and good prescribers (US $11.45 ± 45.0) (Table 1).

The diagnosis with the highest percentage of antibiotic-containing encounters was dental caries in both poor prescribers (91.8%) and good prescribers (86.0%). We identified this indicator to be 23.1% in poor precribers vs. 9.0% in good prescribers (Δ = 156.7%) for the diagnosis of ‘general examination’. The highest difference between the two groups in terms of the antibiotic-containing encounters was found for headache prescriptions (Δ = 899.3%) (Table 1).

We found significantly higher percentages of guideline-compliant drugs for hypertension, tonsillopharyngitis, and acute sinusitis in good prescribers compared to that in poor prescribers (Table 2). The percentage of hypertension prescriptions containing ‘contraindicated’ combination of angiotensin-converting-enzyme inhibitor (ACEi) and angiotensin receptor blocker (ARB) was 0.7% (n = 478) in poor prescribers and 0.5% (n = 232) in good prescribers.

**Table 2.** The comparison of the prescriptions’ compliance to treatment guidelines for disease-based diagnoses by the study groups

		Poor prescribers		Good prescribers		
Indications and guideline compliance		n	%	n	%	p
**Hypertension**	**Guideline-compliant drugs**	24 577	34.8	19 629	40.8	**<0.001**
	**Others**	45 996	65.2	28 534	59.2	
	**Total**	70 573	100.0	48 163	100.0	
**Tonsillopharyngitis**	**Guideline-compliant drugs**	14 579	50.7	26 868	57.9	**<0.001**
	**Others**	14 186	49.3	19 548	42.1	
	**Total**	28 765	100.0	46 416	100.0	
**Acute sinusitis**	**Guideline-compliant drugs**	7,746	43.6	5,408	46.4	**<0.001**
	**Others**	10 019	56.4	6,236	53.6	
	**Total**	17 765	100.0	11 644	100.0	

As treatment guidelines do not directly indicate any drugs for either ‘general examination’ or ‘dental caries’, we accepted the percentage of the guideline-compliant drugs in these prescriptions to be zero. However, the use of symptomatic drugs, for example analgesics, might be justifiable for dental caries. Accordingly, such drugs in dental caries were written by 8.2% (n = 259) of poor prescribers and 13.9% (n = 682) of good prescribers (*P* < 0.001).

**Figure 2.**
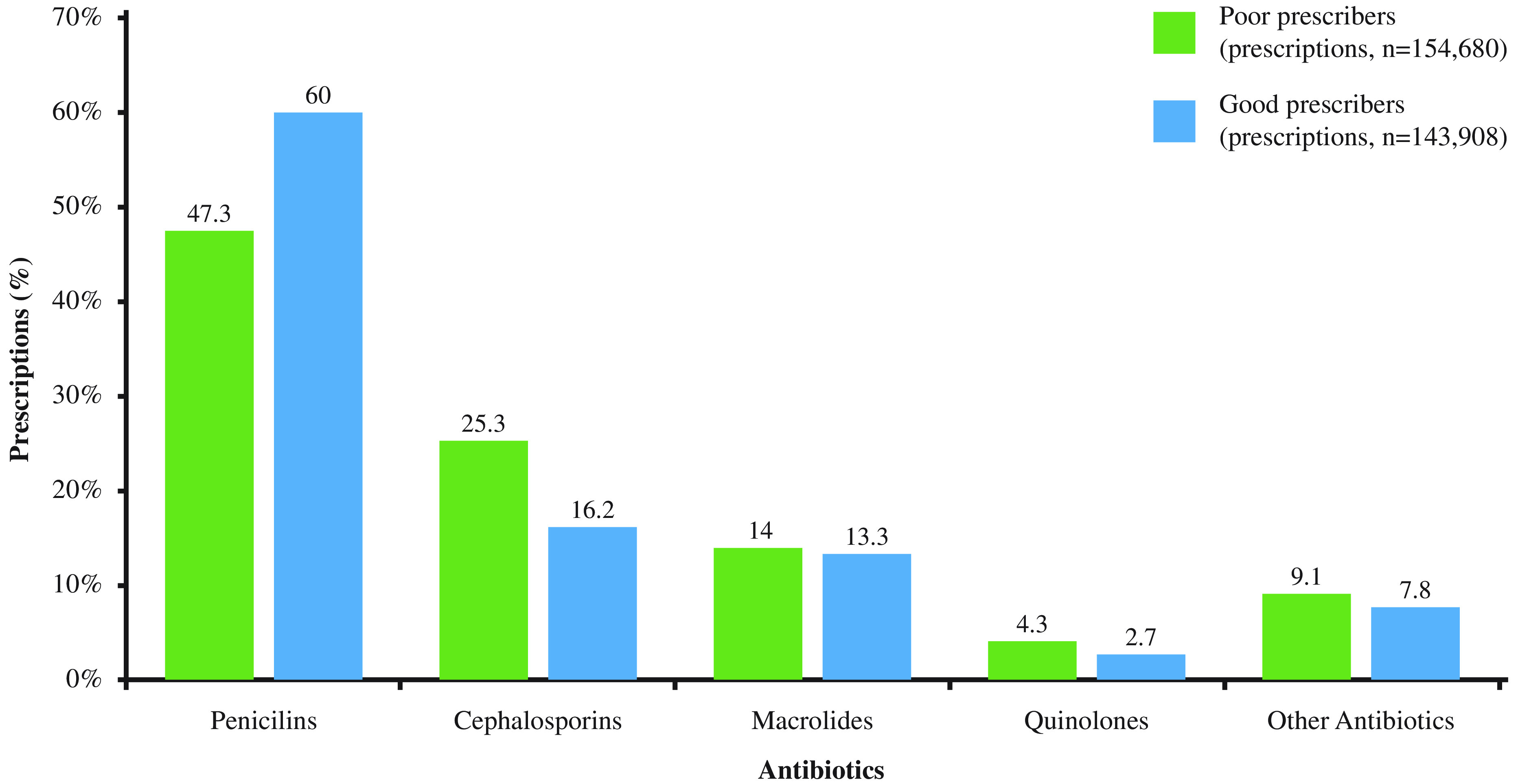
The distribution of antibiotic subgroups in prescriptions of the study groups.

The ranking of the commonly prescribed antibiotic groups was the same in both PCP groups. The most commonly prescribed antibiotics were penicillins (47.3% in poor prescribers and 60.0% in good prescribers), followed by cephalosporins as 25.3% and 16.2%, respectively. Quinolones constituted 4.3% (poor prescribers) and 2.7% (good prescribers) of all antibiotics (Figure 2). In particular, amoxicillin–clavulanate formed 39.5% and 46.2% of all antibiotics, respectively.

For the other drug groups, we found the percentages of PPI-containing prescriptions as 7.1% in poor prescribers and 5.2% in good prescribers. These were 15.3% vs. 14.5% for NSAIDs and 7.4% vs. 5.8% for cough suppressants, respectively.

## Discussion

In this study, we compared the prescribing competencies of PCPs with opposing overall drug use indicators by focusing on the rationality of their pharmacotherapy practice in commonly encountered indications in the community. While the difference between these poor and good performing PCPs was preserved in all diagnoses examined, this gap tended to increase up to about 4-fold in cost and up to 10-fold in antibiotic prescribing. On the other hand, we observed that even more competent physicians seem to remain below the expected performance in some rationality indicators, especially prescribing in compliance with treatment guidelines.

Hypertension is a relatively prevalent condition of primary care, affecting one of every three adults (Kılıçkap *et al.*, 2018; The Ministry of Health of Turkey, 2018). Therefore, it may serve as a cornerstone for the steps toward the detection and the improvement of rationality problems. In our study, the guideline-compliant antihypertensive pharmacotherapy was higher in good prescribers but remained around 40% even in this group. This indicates the need for alternative pharmacological options in the management of hypertension in primary care. In fact, polypharmacy is a frequent practice for this indication (Aubert *et al.*, 2016). This is further supported by the average number of drugs per encounter, varying 2.0 to 2.4 in the study population. However, it is remarkable that such 20% increased tendency of polypharmacy in poor prescribers showed a reflection of 3.8-fold escalation for the mean cost of prescriptions. Another critical approach in the rational management of hypertension is potential vital drug–drug interactions (Mancia *et al.*, 2013). Accordingly, the fact that contraindicated co-prescribing of ACEi and ARB is noticeably observed in both groups with a 41% increased rate in poor prescribers is another dramatic finding of this study. The presence of such irrational co-prescribing practice despite long-time recognition and/or contraindication against their concomitant use in hypertension suggests that there may be a room for further improvement in the adoption of current guidelines by PCPs, especially prioritizing patient safety. Attempts to ensure up-to-datedness of the information about such prevalent indications could promote more rational pharmacotherapy practices in primary care. For example, a UK study examining prescriptions between 2009 and 2015 reported a 19% reduction in the number of prescriptions with co-prescribed renin–angiotensin–aldosterone system blockers within a year following an official warning against their use by the EU in 2014 (Allen & Donegan, 2017; European Medicines Agency, 2014). Such interventions might prevent further harm to patients, focusing on safety and suitability components of RUM. In particular, the latter component of drug selection process during prescribing comprises of many criteria including contraindications, drug interactions, use of drugs in certain populations or indications, emphasizing contribution of patient-oriented pharmacotherapy to RUM.

Irrational use of antibiotics has many negative consequences, including accelerating the antimicrobial resistance, which was identified as one of the ten threats to global health by WHO (World Health Organization, 2019). Antibiotic consumption in Turkey was reported as the highest across the Europe (Versporten *et al.*, 2014). Though more pronounced in poor prescribers, we observed an excessive use of antibiotics being present in >70% of the both tonsillopharyngitis and acute sinusitis prescriptions for both PCP groups. Contrarily, the incidence of bacterial origin in acute sinusitis was reported to vary between 2 and 10% (Chow *et al.*, 2012). There appears a similar situation in tonsillopharyngitis: antibiotics are only indicated for Group A beta-hemolytic streptococcal infections, which was reported as 15%–30% in children and <5% in adults (Shulman *et al.*, 2012). Another study of 2012 reported that PCPs prescribed antibiotics in 80% of all tonsillopharyngitis cases in Turkey (İşli *et al.*, 2017). In this aspect, it can be stated that the use of antibiotics is still far beyond to be rational, especially for these infectious diseases frequently encountered in primary care. In fact, while good prescribers performed better, it appears that only half of the antibiotics prescribed for these two indications are compliance the treatment guidelines in both PCP groups.

The differences observed within the antibiotic prescribing pattern in primary care have become more apparent in the symptom-based diagnoses examined in the study. We determined that poor prescribers wrote antibiotics 3–4-fold in cough and nausea/vomiting and 10-fold in headache than did good prescribers. Although these symptoms can be a part of the clinical manifestations in some infectious diseases, preferring a symptom-based diagnosis to prescribe antibiotics rather than the actual disease itself suggests that irrational behaviors may also be a component of diagnosis process. This is supported by the fact that selecting of ‘general examination’ in the diagnosis section of the prescriptions ranked the 7th and 11th overall in PCP groups. Moreover, we observed >2-fold difference against poor prescribers in terms of both cost and antibiotic use in ‘general examination’ indication. This ambiguous ground for the diagnostic process may not only make it difficult to examine the underlying reasons of irrationality gap but may further complicate the actual assessment regarding the negative prescribing aspects of the poor prescribers, implying a potential vicious cycle. Prescriptions of antibiotic subgroups showed predominance of beta-lactam antibiotics such as penicillins and cephalosporins in both groups. Recently, the use of quinolones has been attempted to be limited as many health authorities recommended against their use for the treatment of uncomplicated infections due to many safety problems (European Medicines Agency, 2018). In our study, we found quinolone use 1.5-fold higher in poor prescribers (4.3%) than that in good prescribers (2.7%). This emphasizes that quantitative difference we observed in irrational antibiotic prescribing overall might be also present in its qualitative aspect, as shown by subgroup distribution of antibiotics, especially for quinolones.

It is thought provoking to see that dental caries, where primary therapy is dental procedures and prescribing is controversial, got so much prescription by PCPs and it was the worst among other diagnoses in terms of compliance to treatment guidelines (SDCEP, 2011). Furthermore, poor prescribers performed worse in all of the WHO/INRUD indicators that concern this diagnosis, especially antibiotics. In some studies, dental conditions are among the reasons for the common use of antibiotics in primary care (Cope *et al.*, 2016; Prah *et al.*, 2017). A recently conducted study about the prescriptions of antibiotics among dentists in Turkey indicated that more than one-third of the antibiotics were prescribed for dental caries (Koyuncuoglu *et al.*, 2017). The pharmacotherapy practice in dental caries detected in our study population should not be addressed only in terms of the poor versus good prescriber difference. Nine out of 10 prescriptions in both groups contain antibiotics, suggesting that the excessive use of antibiotics in dentistry could have a negative impact on primary care. Further studies that will cover dentistry and primary care practice together may help to determine the underlying causes of this issue and contribute to the improvement of dental RUM.

The results of our study should be interpreted by considering its limitations. We evaluated the rationality of pharmacotherapy with the assumption that the diagnoses established by physicians were correct. The underlying possible causes such as comorbidity and antibiotic resistance leading to these diagnoses were not investigated. Poor and good prescribers were determined by examining prescriptions containing all the solo/multiple diagnoses. In order to better evaluate the drug-indication relationship, the study was carried out on solo diagnosis prescription. Therefore, multiple diagnoses prescriptions that were not included in the study may have affected the observed rational prescribing performance of these two groups. However, since the study sample was chosen by randomization and all prescriptions were taken into consideration when determining the poor and good prescribers, the indirect effect in question is thought to be quite weak.

In conclusion, this study shows that the prescribing performances of PCPs are not rational enough in terms of drug selection and prescription content. It is noteworthy that the groups of physicians, which we described as poor and good in terms of general prescribing indicators, show highly heterogeneous prescribing performance in the details regarding the treatment of common diagnoses in primary care. Although this gap between the two groups has been increased disadvantageously for poor prescribers in some indicators, it is understood that even the physicians who have relatively good performance are not satisfactorily rational in compliance to the guidelines and current pharmacotherapy competencies. These findings reveal the necessity of the RUM improvement attempts in accordance with the current national action plan on RUM, which was recently reported to yield successful outcomes in terms of substantial reduction of antibiotic use in primary care (Aksoy *et al.*, 2021). Refinement and/or adaptations of such interventions, especially for the most common indications with a prioritization of poorly performing physicians, could help to further improve RUM in primary care.

## Supplementary material

For supplementary material accompanying this paper visit http://doi.org/10.1017/S1463423622000111. click here to view supplementary material
