# Supplementary material for: Good versus poor prescribers: the comparison of prescribing competencies in primary care
Source: Prim Health Care Res Dev. 2022 Mar 28;23:e22. doi: 10.1017/S1463423622000111 (PMC8991858; doi:10.1017/S1463423622000111)
Supplement: Supplementary file 1 [file S1463423622000111sup001.docx]

**SUPPLEMENTARY MATERIAL**

**Supplementary Table S1.** The distribution of selected drug utilization criteria by the quartiles of the primary care physicians in all prescriptions.

| **Drug utilization criteria** | **1^st^ Quartile (n=210)** | **2^nd^ Quartile (n=203)** | **3^rd^ Quartile (n=199)** | **4^th^ Quartile (n=227)** |
| --- | --- | --- | --- | --- |
| **Average number of medicines per encounter, n (± SD)** | 2.39±0.14 | 2.74±1.12 | 2.55±0.78 | 3.22±2.24 |
| **Encounters with an antibiotic prescribed, %** | 21.1 | 19.9 | 23.2 | 26.5 |
| **Average number of drug boxes per encounter, n (±SD)** | 4.57±0.71 | 5.85±1.20 | 5.12±0.90 | 7.92±2.34 |
| **Average medicine cost per encounter, US$ (±SD)** | 26.19±5.30 | 35.13±11.94 | 32.61±7.49 | 48.37±38.04 |

**Supplementary Table S2.** Descriptive characteristics of drug use indicators in study groups for solo-diagnosis prescriptions.

|  | **Poor prescribers (n=227)** | **Good prescribers (n=210)** |
| --- | --- | --- |
| **Total number of prescriptions, n (%)*** | 550,054 (18.7) | 656,879 (22.3) |
| **Average number of medicines per encounter, n (± SD)** | 2.38±1.20 | 1.92±0.24 |
| **Encounters with an antibiotic prescribed, %** | 28.3 | 23.1 |
| **Average number of drug boxes per encounter, n (±SD)** | 4.70±1.32 | 3.23±0.45 |
| **Average medicine cost per encounter, US$ (±SD)** | 32.87±15.71 | 16.96±3.27 |

* Percentage among solo-diagnosis prescriptions written by 1,431 physicians.

**Supplementary Table S3.** The distribution of study-defined diagnoses established by the study groups with their ranks and coverage in the prescriptions of the respective group.

| **Diagnoses** | **Poor prescribers (n=227)** | | | **Good prescribers (n=210)** | | |
| --- | --- | --- | --- | --- | --- | --- |
|  | Rank | Prescriptions,  n (%) | Physicians,  n (%) | Rank | Prescriptions,  n (%) | Physicians,  n (%) |
| **Hypertension** | 1. | 70,573 (12.8) | 212 (93.4) | 2. | 48,163 (7.3) | 200 (95.2) |
| **Tonsillopharyngitis** | 2. | 28,775 (5.2) | 206 (90.8) | 3. | 46,416 (7.1) | 203 (96.7) |
| **Acute sinusitis** | 10. | 17,765 (3.2) | 203 (89.4) | 6. | 11,644 (1.8) | 195 (92.9) |
| **General exam** | 11. | 8,720 (1.6) | 191 (84.1) | 7. | 15,768 (2.4) | 181 (86.2) |
| **Dental caries** | 41. | 3,159 (0.6) | 110 (48.5) | 26. | 4,910 (0.7) | 118 (56.2) |
| **Dorsalgia** | 13. | 8,088 (1.5) | 194 (85.5) | 12. | 10,978 (1.7) | 183 (87.1) |
| **Dyspepsia** | 19. | 3,969 (0.7) | 160 (70.5) | 32. | 4,113 (0.6) | 142 (67.6) |
| **Cough** | 33. | 2,712 (0.5) | 100 (44.1) | 31. | 4,865 (0.7) | 136 (64.8) |
| **Nausea and vomiting** | 43. | 2,347 (0.4) | 184 (81.1) | 41. | 3,579 (0.5) | 185 (88.1) |
| **Headache** | 44. | 1,798 (0.3) | 123 (54.2) | 49. | 2,744 (0.4) | 149 (71.0) |
| **Subtotal** |  | 147,906 (26.9) | - |  | 153,180 (23.3) | - |
| **Others** |  | 402,148 (73.1) | - |  | 503,699 (76.7) | - |
| **Total** |  | 550,054 (100.0) | - |  | 656,879 (100.0) | - |
